# Long-term methimazole therapy in Graves’ hyperthyroidism and adverse reactions: a Danish multicenter study

**DOI:** 10.1530/ETJ-22-0031

**Published:** 2022-04-22

**Authors:** J Karmisholt, S L Andersen, I Bulow-Pedersen, A Krejbjerg, B Nygaard, A Carlé

**Affiliations:** 1 Department of Endocrinology, Aalborg University Hospital, Aalborg, Denmark; 2 Department of Clinical Institute, Aalborg University, Aalborg, Denmark; 3 Department of Clinical Biochemistry, Aalborg University Hospital, Aalborg, Denmark; 4 Department of Oncology, Aalborg University Hospital, Aalborg, Denmark; 5 Department of Endocrinology and Internal Medicine, Herlev University Hospital, Copenhagen, Denmark

**Keywords:** hyperthyroidism, thyrotoxicosis, Graves’ hyperthyroidism, Graves’ disease, TSH-receptor anti-bodies, anti-thyroid drugs adverse drug reactions, adverse events

## Abstract

**Purpose:**

In this prospective multicenter study with patients newly diagnosed with Graves’ hyperthyroidism (GH), we studied the timing and characteristics of adverse drug reactions in patients treated with anti-thyroid drugs (ATD) for up to 48 months.

**Methods:**

Patients with GH were treated with ATD until remission and hereafter with a low-dose regime to keep the patients in remission. The patients were followed with blood samples and recording of adverse events approximately every second month for the first 2 years and every third month for the following 2 years.

**Results:**

We included 208 patients and the patients were treated for a median of 22 (range: 0.5–49) months. Ten percent of the patients experienced adverse drug reactions and 75% of the cases occurred during the first 6 months. After 24 months, the methimazole dose was lowered to 5 mg/day, and after this time point, no further adverse drug reactions were recorded. Skin reactions were the most prominent reaction, comprising 68% of the registered reactions, and no hepatic and bonemarrow affection was recorded.

**Conclusion:**

With this study, we report the frequency, timing of occurrence, and characteristics of adverse drug reactions when treating GH with the ATD drug methimazole for up to 48 months. Long-term low-dose methimazole treatment can be a cost-effective and straightforward treatment option if adverse drug reactions such as severe hepatic and bone marrow affection are kept in mind.

## Introduction

Graves’ hyperthyroidism (GH) is an autoimmune disease mainly affecting the thyroid gland (1, 2). The disease is usually transient with remission occurring within a period of 1–2 years after treatment with anti-thyroid drugs (ATD). However, relapse is frequent and seen in around 50% of cases (3, 4, 5). The unpredictable course is probably one of the reasons for the differences in treatment strategies recommended and used by different thyroid societies (6, 7, 8). As around 50% of the patients achieve and sustain remission after 12–18 months of ATD treatment, the use of this treatment strategy has been increasing recently (9). The main advantage of using ATD in GH is the fact that this treatment does not destroy thyroid tissue and once remission is achieved, many patients continue to have normal thyroid function without any treatment. Radioactive iodine or surgery, on the other hand, almost inevitably leaves the patients with a lifelong need for L-T4 substitution therapy. The more favorable adverse drug reaction (ADR) profile of L-T4 substitution therapy compared to the drug reaction profile of ATDs favors this strategy. However, the increased focus on the reduced quality of life in patients on L-T4 substitution therapy raises doubts about this claim (10, 11). Thus, long-term ATD in GH has become an increasingly used treatment option in recent years (12).

‘Remission Induction and Sustenance in Graves’ Disease (RISG)’ was a multicenter study that aimed to improve the knowledge of how patients with GH enter remission during ATD therapy and to evaluate if remission could be sustained in a subgroup of patients by a more prolonged low-dose ATD therapy strategy (13). In the RISG study, patients with GH were treated with ATD for up to 48 months. We have previously shown that TRAb measured at the time of diagnosing GH was a prognostic marker of attaining remission and that consecutive TRAb measurements during treatment were not worthwhile and in patients in remission, only 3.6% experienced relapse during 24 months of follow-up when treated with a fixed dose of 5 mg methimazole (MMI) in combination with L-T4 supplementation (14).

In the present analysis, we aimed to examine the timing and characteristics of ADRs in patients with GH who were treated with ATD for up to 48 months. We hypothesized that long-term ATD drugs could be favorable and with few side effects, and thus an alternative treatment strategy to destructive therapy with surgery or radioiodine, in a subgroup of patients.

## Materials and methods

The inclusion process, treatment modality, and description of remission and relapse have previously been published (13, 14). We included patients with GH (total T3 above the upper limit of normal (ULN) and suppressed thyrotropin (TSH) and thyroid-receptor antibody (TRAb) positivity or diffuse uptake on thyroid scintiscan) who did not receive treatment for hyperthyroidism the previous 2 years. Patients were recruited from two Danish centers, the Departments of Endocrinology at Aalborg University Hospital and Herlev University Hospital, Copenhagen. The patients were included from January 11, 2007, to June 6, 2011. Exclusion criteria were: age <18 years, pregnancy, moderate to severe orbitopathy with a need of medical immunosuppressive therapy, intake of drugs affecting the immune system, imminent or manifest thyrotoxic crisis, other severe disease making it unlikely for the patient to be able to follow the protocol, thyroid nodules necessitating surgery, intolerance to both MMI and propylthiouracile (PTU), and previous surgical or radioiodine therapy for Graves’ disease.

The RISG study was approved by the local ethical committee North Denmark Region Committee on Health Research Ethics (VN-20060062) and registered in ClinTrial.gov (NCT00796913). All the patients provided informed consent.

**Figure 1:**
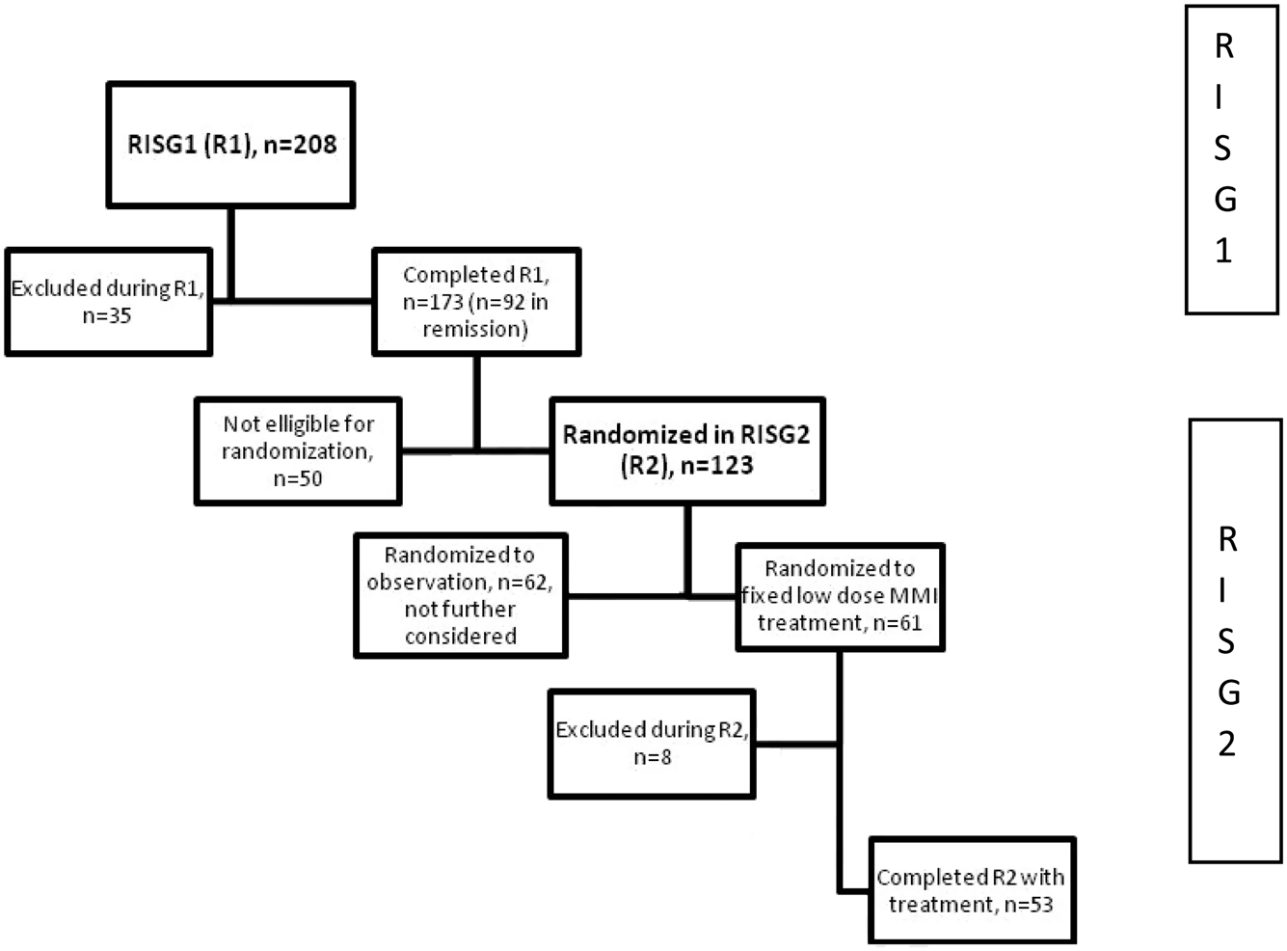
Flowchart of the two sequential sub-part of the RISG study. Reasons for exclusion in the two sequential sub-parts were: in R1: lack of compliance (*n*  = 15), development of orbitopathy (*n*  = 7), moving out of the area (*n*  = 4), ADR (*n*  = 4), comorbidities (*n*  = 3), pregnancy (*n*  = 1), and suspicion of thyroid cancer (*n*  = 1); in R2: withdrawal of consent (*n*  = 3), pregnancy (*n*  = 2) lack of compliance (*n*  = 1), joint pain (*n*  = 1), and death (*n*  = 1).

The RISG study comprised two sub-parts, RISG1 (treatment until remission) followed by RSIG2 (randomization to just observation or fixed low-dose ATD), which is illustrated in Fig. 1. Fixed low-dose ATD was MMI 5 mg/day in combination with L-T4 of 1 µg/bodyweight in kilogram/day. The patients were followed until remission or for 24 months (RISG1) and after this, the patients were followed until they relapsed or for additional 24 months (RISG2). In the study, remission was pre-specified and defined as TSH >0.4 mU/L and TRAb ≤1. 0 IU/L in a patient receiving an MMI dose ≤5 mg/day, on two occasions 2 months apart. Relapse was defined by TSH <0.01 mU/L and total T3 ≥3.0 nmol/L combined with TRAb ≥1.0 IU/L.

In the RISG1 sub-part, the patients were investigated at baseline and after 2–3 weeks, 5–6 weeks, 2, 3, 4 months, and every second month from there up to 24 months. In the RISG2 sub-part, the patients were investigated at baseline and after 1, 3, 6, 9, 12, 18, and 24 months. Each investigation comprised blood samples, possible adjustment of ATD dose, and communication with the patient regarding thyroid hormone levels, treatment, and ADRs. At baseline, when changing from the RISG1 to the RISG2 part and after completing either of the two sub-parts, thyroid ultrasound and clinical examination were added to the investigation, as well as blood samples with liver function tests, hematological parameters, and anti-neutrophil cytoplasmic antibody (ANCA) measurements. As part of the clinical examination, the responsible clinician was asked to register any symptoms and signs of potential ADR including skin reactions, vasculitis, agranulocytosis, and liver failure.

### Thyroid hormone and antibody assays

Serum total T3 (reference range: Aalborg 1.1–2.5 nmol/L, Copenhagen 1.0–2.6 nmol/L), and TSH (reference range: Aalborg 0.30–4.5mU/L, Copenhagen 0.40–4.0 mU/L) were measured by automatic routine laboratory immunoassays (Roche Diagnostics Elecsys; Immulite 2500, Siemens). TRAb was measured using a manual, competitive, second-generation RIA (DYNOtest TRAK, Thermo Fisher). TRAb ≥ 1.0 IU/L was considered as TRAb positivity (15).

### Statistics and calculations on the cost of treatment

Data were analyzed using IBM SPSS statistics version 26.0. Student’s *T*-test was used for comparisons between groups and chi-square or Fisher’s exact test was used for categorical data. For time-to-event, the Kaplan–Meyer survival estimation method was performed (16). A *P*-value < 0.05 was considered statistically significant.

For calculations on the cost of treatment, we used the official Danish diagnosis-related group rates (https://interaktivdrglpr2.sundhedsdata.dk/) and drug price (https://pro.medicin.dk/Medicin/Indholdsstoffer/628, https://pro.medicin.dk/Medicin/Indholdsstoffer/995). The cost of surgery was set to €6000 (Euros), radioiodine treatment to €1000, cost of a daily dose of MMI to €0.20, and L-T4 to €0.13. In the calculation, the cost of monitoring the treatment was not included (and not expected to differ between the three treatment strategies), and it was assumed that lifelong L-T4 substitution therapy was warranted following surgery or radioiodine treatment and that all patients live until the age of 85.

## Results

We included 208 patients in the RISG1 sub-part, and 123 patients were eligible for randomization in the RISG2 sub-part (Fig. 1). In RISG1, 35 patients had an adverse event prompting exclusion from the study. Fifteen patients were excluded due to a lack of compliance with the protocol. The main reason for this was the many blood samples required at certain time points. Three patients were excluded due to co-morbidities: one due to pre-existing autoimmune hepatitis, one had pre-existing severe heart failure, and a third patient was diagnosed with progressive dementia. The remaining 17 patients were excluded due to orbitopathy (*n*  = 7), moving out of the area (*n*  = 4), ADR to ATD (*n*  = 4), pregnancy (*n*  = 1), and observation for thyroid cancer (*n*  = 1). In the RISG2 sub-part, eight patients were excluded due to withdrawal of consent (*n*  = 3), lack of compliance with the protocol (*n*  = 1), joint pain (*n*  = 1), pregnancy (*n*  = 1), and death (*n*  = 1). The cause of death for the patient who died during the study was cholangiocarcinoma. This patient had alkaline phosphatase at 157 U/L (normal range: 35–105U/L) at randomization (RISG2). Otherwise, all alanine aminotransferase, alkaline phosphatase, and bilirubin measurements were normal and the development of cholangiocarcinoma was not attributed to the MMI treatment.

Overall, the median duration of MMI treatment in the patients was 22 months, with a range of 0.5–49 months and 25 and 75 percentiles of 10 and 31 months.

**Figure 2:**
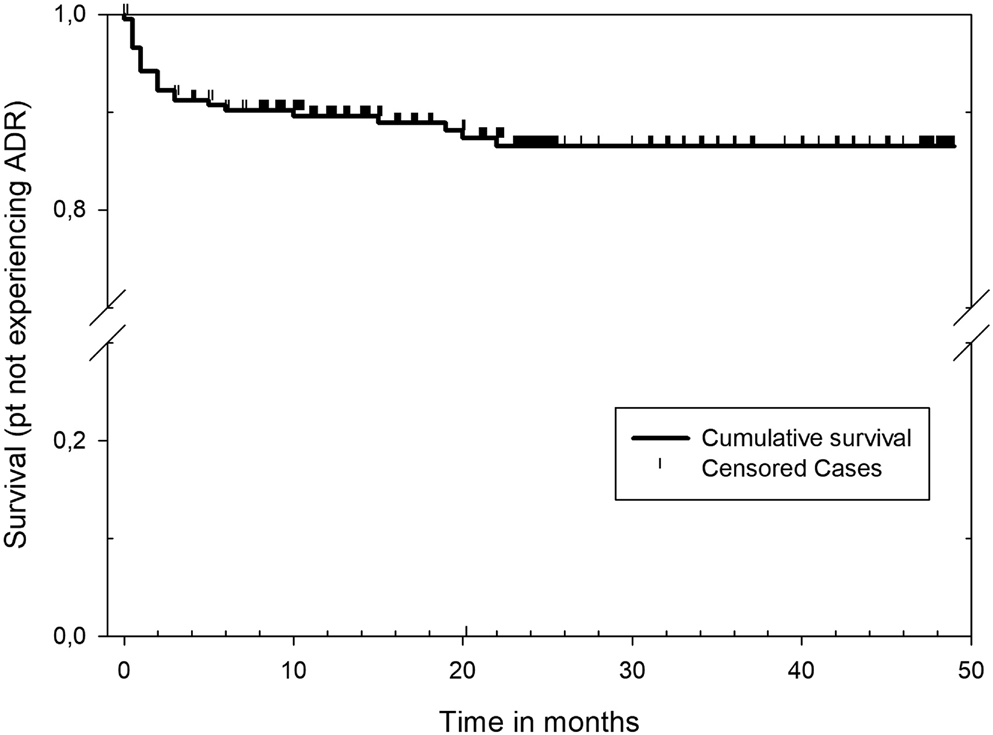
A Kaplan–Meyer plot of the occurrence of ADRs during methimazole treatment.

Twenty-five patients experienced ADRs, of which four were graded as severe prompting exclusion from the study. There were no significant differences between the patients with or without ADRs in any of the investigated baseline parameters (Table 1). In Table 2, it is shown that skin reactions were the first to develop and the most predominant adverse reaction and constituted two-thirds of the reactions. Other reactions were joint reaction (*n*  = 3), gastrointestinal symptoms (*n*  = 2), hair loss (*n*  = 1), muscle cramps (*n*  = 1), and periorbital itching and discomfort (*n*  = 1), not suspected to represent Graves’ orbitopathy. The initial response to an ADR was to change ATD from MMI to PTU, which was done in all, but two, cases. No severe hepatic or bone marrow affection was observed, neither was vasculitis nor pancreatitis. Overall, around 10% of the treated patients experienced ADRs and most reactions occurred during the initial months of treatment, as 50% of the ADRs were registered after 2 months and 75% after 6 months of treatment (Fig. 2 and Table 2). After 24 months of treatment, a maximum dose of MMI 5 mg/day only was used, and after this time point, no ADRs were registered apart from the only patient who was treated with PTU and L-T4, in whom joint symptoms subsided when the L-T4 drug was changed.

**Table 1:** Baseline characteristic of patient with Graves’ hyperthyroidism experiencing adverse drug reaction (ADR) to anti-thyroid drug (ATD) compared to patients with no ADRs.

	No ADR	ADR	*P*-value of difference^a^
	Mean (s.d.)	Mean (s.d.)	
Number	183	25	
Age, years	44.6 (13.8)	43.8 (9.41)	0.794
Sex (%, male)	16.4	8.0	0.382
BMI (kg/m^2^)	24.1 (4.77)	23.3 (4.85)	0.405
GO (mild + moderate, %)	30.6	24.0	0.643
Smoking (yes, %)	26.2	32.0	0.631
S-TRAb, IU/L	17.1 (38.1)	14.5 (16.5)	0.738
S-T3, nM	6.2 (3.0)	6.1 (2.8)	0.870
Thyr Vol, mL	25.2 (17.4)	23.0 (12.1)	0.556
ANCA normal, %	97.6	100	0.630
ALAT normal, %	65.9	64.0	0.506
Initial MMI dose, mg/day	22.5 (10.3)	25.5 (15.2)	0.214

^a^T-test/Chi-squared or Fischer’s test.

ALAT, alanine aminotransferase; ANCA, anti-neutrophil cytoplasmic antibody; GO, Graves’ orbitopathy; MMI, methimazole; T3, triiodothyronin; Thyr Vol, thyroid gland volume measured by ultrasound; TRAb, TSH-receptor antibody.

**Table 2:** A list of occurrence and timing of all recorded adverse drug reactions (ADR) to anti-thyroid drugs (methimazole) when treating Graves’ hyperthyroidism for up to 48 months. One patient died of cholangiocarcinoma, and this was recorded as an adverse event and not an ADR attributed to methimazole.

Adverse drug reactions (ADR)	Time in days, to onset (median; range)	Percent of total ADR (%)
Skin reaction	30; 14–743	68
Joint reaction	92; 70–192	12
Muscle cramps	96; n.a	4
Gastrointestinal symptoms	110; 62–157	8
Hairloss	153; n.a	4
Periorbital itching	236; n.a	4

n.a., not applicable.

## Discussion

In this study, ADRs of MMI used for up to 48 months were studied. Around 10% of the treated patients experienced ADR. Of these, 16% were graded as moderate to severe, prompting exclusion from the study due change in treatment strategy to either radioactive iodine or surgery. The most noticeable reaction was skin reactions as this consisted 68% of all reactions. Most ADRs occurred after a few months of treatment, as 75% of the total registered reactions were recorded after 6 months. If MMI was tolerated and the dose was kept at 5 mg/day or lower, no ADRs were recorded when the treatment continued from 24 to 48 months.

ATD has been in clinical use for over 70 years (17). A wide variety of ADRs has been registered (18, 19). The most severe being hepatic and bone marrow affection and vasculitis. In the present study, no new or hitherto unrecognized side effects were observed. Two prospective studies of long-term ATD treatment in GH state the frequency of ADRs. In the study by Chen and co-workers, 230 hyperthyroid patients (174 had GH) were randomized to ATD and 230 to radioactive iodine. In the ATD group, 12% of the patients experienced ADR (skin reactions 57%, liver affection 18%, and agranulocytosis 25%) (20). Azizi and co-worker also recorded ADR in their long-term study of ATD treatment in 302 GH patients (21). In this study, 5.3% experienced ADR (skin reaction 87.5% and liver affection 12.5%). Thus, our study is in line with these two Asian studies both in terms of the overall ADR prevalence and the overwhelming presence of skin reactions in those experiencing any adverse reaction. It is clear from the present data that most ADRs occur during the first months of treatment, and this gives a hint to when follow-up clinical control is relevant in patients treated with ATDs. Our study also showed that if tolerated for 24 months, a low-dose long-term MMI treatment seems to be very well-tolerated and also is associated with very low relapse rates of 3.6% during 24 months of follow-up (14). Thus, patients who have experienced the previous relapse may after 2 years of treatment be encouraged to continue low-dose ATD with MMI.

**Figure 3:**
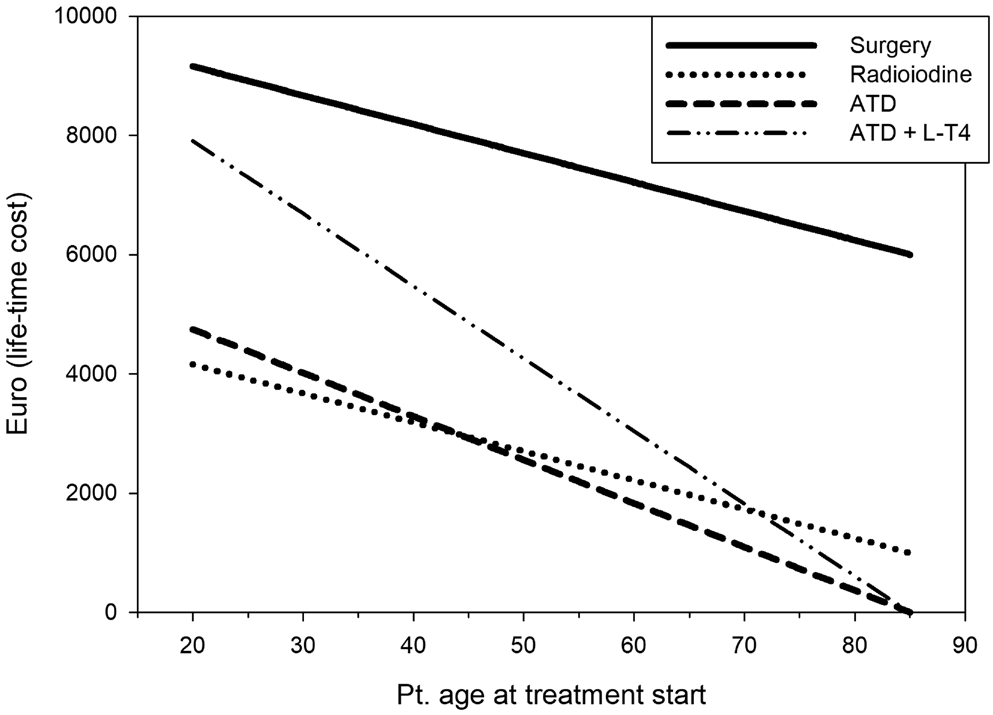
Treatment cost of lifelong treatment for three different treatment modalities of Graves’ hyperthyroidism. The cost of surgery was set to €6000 (Euros), radioiodine treatment to €1000, cost of a daily dose of MMI to €0.20, and L-T4 to €0.13. It was assumed that lifelong L-T4 substitution therapy is warranted following surgery or radioiodine and that all patients live until the age of 85. The cost of monitoring the therapy was not included but was not expected to differ between the three treatment modalities.

The lifetime cost of treating GH with either ATD, surgery, or radioactive iodine was performed and shown in Fig. 3. From Fig. 3, it is evident that radioiodine treatment is the most cost-effective therapy, but after the age of 45 years, ATD is comparable or equivalent in price to radioiodine, and regardless of age, ATD is economically favorable compared to surgery. We thus suggest that in patients with long-lasting GH without goiter or thyroid nodule issues, a low dose of ATD (equivalent to MMI 5 mg/day or lower) is an effective (14), a well-tolerated, and cost-effective treatment choice compared to surgery regardless of age and compared to radioiodine in patients 55 years or older. If L-T4 substitution therapy is added to the ATD regime, the treatment cost will increase, and this strategy is comparable in cost to radioiodine therapy in patients aged 75 years or older.

### Strengths and limitations

In this prospective study, ADRs were recorded consecutively and thus recall bias was minimized. Another strength of the study is the strict and frequent assessment of the patients, making it possible to quite precisely record the timing of events. In this two-center study, inclusion and treatment were tailored after hormone values in blood samples. For this, we used assays measuring total thyroid hormone, as this was used in clinical practice in the two centers at the time of the study. Currently, free hormone measurements are mostly used, but total thyroid hormone measurements are considered more robust (22). As the study included non-pregnant patients and the diagnosis was based on the combination of low TSH, high T3, and TRAb positivity, we do not think the diagnosis of GH was influenced using total T3. We also find it unlikely that monitoring treatment with total T3 could influence the patient’s reaction to ATDs. Only one patient was started on PTU. Hence, the results and the conclusion are only applicable to patients with GD treated with MMI.

Although we included 208 patients with GH, the complex design with two sub-parts, and two study arms in the second part, the number of patients and events was limited. However, we think the conclusion of the study is justified as the patients were seen at quite frequent follow-ups and 64 patients were followed for more than 24 months of treatment.

## Conclusion

With this study, we report the frequency, timing of occurrence, and characteristics of ADRs when treating GH with MMI for up to 48 months. Ten percent of patients treated with ATDs experienced ADRs, with 75% of the cases occurring during the first 6 months of treatment, and skin reactions were the most prominent reaction. If MMI was tolerated during 24 months of treatment, further treatment with MMI of 5 mg/day or lower was in practice without ADRs. Long-term, low-dose MMI treatment can be a cost-effective, safe, and straightforward treatment option if ADRs such as severe hepatic and bone marrow affection are kept in mind.

## Declaration of interest

The authors declare that there is no conflict of interest that could be perceived as prejudicing the impartiality of the research reported.

## Funding

This work did not receive any specific grant from any funding agency in the public, commercial, or not-for-profit sector.
